# Accurate detection of *KRAS*, *NRAS* and *BRAF* mutations in metastatic colorectal cancers by bridged nucleic acid-clamp real-time PCR

**DOI:** 10.1186/s12920-019-0610-8

**Published:** 2019-11-11

**Authors:** Yuki Nagakubo, Yosuke Hirotsu, Kenji Amemiya, Toshio Oyama, Hitoshi Mochizuki, Masao Omata

**Affiliations:** 1 Division of Genetics and Clinical Laboratory, Yamanashi Central Hospital, 1-1-1 Fujimi, Kofu, Yamanashi Japan; 2 Genome Analysis Center, Yamanashi Central Hospital, 1-1-1 Fujimi, Kofu, Yamanashi Japan; 3 Department of Pathology, Yamanashi Central Hospital, 1-1-1 Fujimi, Kofu, Yamanashi Japan; 4 Department of Gastroenterology, Yamanashi Central Hospital, 1-1-1 Fujimi, Kofu, Yamanashi Japan; 5 The University of Tokyo, 7-3-1 Hongo, Bunkyo-ku, Tokyo, Japan

**Keywords:** *KRAS*, *NRAS*, *BRAF*, Colorectal cancer, BNA

## Abstract

**Background:**

Patients with metastatic colorectal cancer can benefit from anti-EGFR therapy, such as cetuximab and panitumumab. However, colorectal cancers harboring constitutive activating mutations in *KRAS*, *NRAS* and *BRAF* genes are not responsive to anti-EGFR therapy. To select patients for appropriate treatment, genetic testing of these three genes is routinely performed.

**Methods:**

We applied bridged nucleic acid-clamp real-time PCR (BNA-clamp PCR) to detect somatic hotspot mutations in *KRAS*, *NRAS* and *BRAF*. PCR products from BNA-clamp PCR were subsequently analyzed Sanger sequencing. We then compared results with those from the PCR–reverse sequence-specific oligonucleotide probe (PCR-rSSO) method, which has been used as in vitro diagnostic test in Japan. To validate the mutation status, we also performed next generation sequencing using all samples.

**Results:**

In 50 formalin-fixed paraffin-embedded tissues, *KRAS* mutations were detected at frequencies of 50% (25/50) and 52% (26/50) by PCR-rSSO and BNA-clamp PCR with Sanger sequencing, respectively, and *NRAS* mutations were detected at 12% (6/50) and 12% (6/50) by PCR-rSSO and BNA-clamp PCR with Sanger sequencing, respectively. The concordance rate for detection of *KRAS* and *NRAS* mutations between the two was 94% (47/50). However, there were three discordant results. We validated these three discordant and 47 concordant results by next generation sequencing. All mutations identified by BNA-clamp PCR with Sanger sequencing were also identified by next generation sequencing. BNA-clamp PCR detected *BRAF* mutations in 6% (3/50) of tumor samples.

**Conclusions:**

Our results indicate that BNA-clamp PCR with Sanger sequencing detects somatic mutations in *KRAS*, *NRAS* and *BRAF* with high accuracy.

## Background

The incidence of colorectal cancer has been increasing. Metastatic colorectal cancers have a high mortality rate with a five-year survival rate of less than 10%. Genetic alterations in RAS–MAPK and PI3K–AKT pathway are common in colorectal cancers. The most recurrently mutated genes in these pathways are *KRAS* (Kirsten rat sarcoma viral oncogene homolog, OMIM: 190070), *NRAS* (neuroblastoma RAS viral oncogene homolog, OMIM: 164790) and *BRAF* (v-raf murine sarcoma viral oncogene homolog B1, OMIM: 164757). Monoclonal antibodies against the epidermal growth factor receptor (EGFR), including cetuximab and panitumumab, have been used to treat patients with metastatic colorectal cancer. These antibodies bind to the extracellular domain of EGFR and inhibit its downstream signaling, which mainly affects cell proliferation and survival via RAS-RAF-MEK-ERK and PI3K-AKT pathways.

Anti-EGFR therapy is beneficial in approximately 15% of patients with wild-type *KRAS* metastatic colorectal tumors, whereas patients with *KRAS*-mutated tumors show little response [1–5]. Furthermore, anti-EGFR therapy is more beneficial to patients with wild-type *NRAS* and *BRAF* [6–8]. It is well-known that somatic hotspot mutations are located in codons 12 and 13 (exon 2), codons 59 and 61 (exon 3), and codons 117 and 146 (exon 4) of *KRAS* and *NRAS* genes, and in codon 600 (exon 15) in *BRAF*. In colorectal cancers, *KRAS* mutations are observed in 42% of cases, while mutations in *NRAS* (10%) and *BRAF* (10%) are less frequent [9, 10]. *KRAS*, *NRAS* and *BRAF* mutations occur in colorectal cancers in a mutually exclusive manner [10].

Genetic analysis of somatic hotspot mutations in *KRAS*, *NRAS* and *BRAF* is now standard practice for selecting patients for anti-EGFR therapies. To simultaneously detect different types of mutations by real-time PCR, we tested the bridged nucleic acid (BNA)-clamp technique [11]. A BNA is an artificial nucleic acid that strongly binds to a complementary DNA structure [12]. BNA-clamp PCR enables mutations to be detected because the melting temperature of a perfectly matched BNA-DNA duplex is much higher than that of DNA-DNA duplex [13–15]. Furthermore, mutated alleles can be selectively amplified because the BNA clamp oligonucleotide inhibits amplification of the wild-type allele.

In this study, we examined the clinical utility of the BNA-clamp PCR technique to detect *KRAS*, *NRAS* and *BRAF* mutations. To this end, we determined the mutation status of 50 patients with colorectal cancer from formalin-fixed paraffin embedded (FFPE) tissues and compared these results with those from the PCR–reverse sequence-specific oligonucleotide probe (PCR-rSSO) method, which is approved for in vitro diagnostic test for analyzing *KRAS*, *NRAS* and *BRAF* in patients with colorectal cancer in Japan [16]. To validate the mutations status in three genes, we conducted panel sequencing by next generation sequencing (NGS) [17–27].

## Methods

### Samples and study design

We collected tumor tissues from 50 patients with colorectal cancer between November 2010 and February 2016 and prepared FFPE samples from these tissues. The samples were analyzed by SRL Inc. (Tokyo, Japan) using an in-vitro diagnostic PCR-rSSO kit. We analyzed the same tumor samples by the BNA-clamp method.

To estimate the concordant and discordant results were obtained by the two methods, validation was performed by next generation sequencing (NGS) analysis using an Ion PGM system (Thermo Fisher Scientific, MA, USA) [17, 18, 24, 28]. The Institutional Review Board of clinical research and genome research committee at Yamanashi Central Hospital approved this retrospective study and written informed consent was obtained from patients. Patients had the opportunity to refuse to participate in the study.

### PCR-rSSO

Five 10-μm thick sections from each of the 50 FFPE tissues were analyzed by PCR-rSSO using a MEBGEN™ RASKET kit (MBL, Nagoya, Japan) [29, 30]. This analysis was performed by SRL Inc. (Tokyo, Japan). The PCR-rSSO kit detected 48 types of mutation in *KRAS* and *NRAS*, but did not target *BRAF*. In brief, multiplex PCR amplified codons 12, 13, 59, 61, 117 and 146 in *KRAS* and *NRAS* using eight sets of biotinylated primer pairs. PCR products were hybridized with complementary mutated probes immobilized on fluorescent-beads. After washing, the hybridized beads were mixed with phycoerythrin-labeled streptavidin (SA-PE) solution. Fluorescence was detected on a Luminex100/200 instrument (Luminex) and the types of mutation Identified.

### BNA-clamp PCR

For each of the 50 samples, DNA was extracted from two 10-μm thick FFPE sections and from five 10-μm thick tumor biopsy sections using an Agencourt FormaPure DNA kit (Beckman Coulter, CA, USA) according to the manufacturer’s protocol. DNA concentration was determined using a NanoDrop 2000 spectrophotometer (Thermo Fisher Scientific). If tumor purity was less than 10%, we performed laser capture microdissection to enrich for tumor cells. To this end, tumor tissues were stained with hematoxylin and eosin, and then microdissected using an ArcturusXT laser capture microdissection system (Thermo Fisher Scientific).

To detect mutations in *KRAS*, *NRAS* and *BRAF*, we used a BNA Real-time PCR Mutation Detection Kit Extended RAS (Riken Genesis, Tokyo, Japan). This kit contains nine types of BNA-probe, primers and PCR enzymes for detecting mutations by quantitative real-time PCR. Nine types of primer/probes were designed in house to target *KRAS* at codons 12/13, 59/61, 117 and 146, *NRAS* at codons 12/13, 59/61, 117 and 146, and *BRAF* at codon 600. The BNA is an artificial nucleic acid that hybridizes to a perfectly matched template with high affinity. A BNA clamp selectively inhibits PCR of the wild-type template, but does not influence a mutated template [12]. According to the manufacturer’s protocol, the reaction mixture comprised 12.5 μL 2x Master Mix, 2.5 μL 10x Oligo mix, 0.4 μL 25 μM ROX™ Reference Dye, 0.25 μL Uracil-N-glycosylase (UNG), and 20–100 ng FFPE DNA in a 25 μL total volume. Real-time PCR was conducted on a ViiA7 Real Time System (Thermo Fisher Scientific) with the following cycling conditions: 50 °C for 3 min, 95 °C for 2 min, and 40 cycles of 95 °C for 30 s and 60 °C for 45 s*.* The data were analyzed using ViiA7 software v2.2.2 (Thermo Fisher Scientific). The threshold line was set at 0.04. The threshold cycle (Ct) value was assigned to each PCR reaction and amplification curve was visually assessed. When amplification plot did not reach to threshold line, we examined whether the sample harbored mutations by Sanger sequencing using BNA-clamp PCR products (Additional file 4: Table S1).

### Sanger sequencing

To further determine nucleotide changes and to characterize the deduced amino acid changes, we performed Sanger sequencing on samples, in which PCR amplification plot was observed but did not reached to threshold line by BNA-clamp PCR. BNA-clamp PCR products were purified using ExoSAP-IT Express PCR Cleanup Reagent (Thermo Fisher Scientific) [31, 32]. Purified products were used as templates and Sanger sequencing was performed using the BNA Real-time PCR Extended RAS Mutation Sequencing Primer (Riken Genesis) and the BigDye® Terminator v3.1 Cycle Sequencing Kit (Thermo Fisher Scientific). Sequencing reactions consisted of 1.0 μL template PCR product, 0.5 μL 3.2 μM forward primer or 0.5 μL 3.2 μM reverse primer, 2 μL Big Dye Buffer, 1 μL BigDye v3.1 and 5.5 μL nuclease free water. PCR was conducted on a Veriti Thermal Cycler (Thermo Fisher Scientific) with the following cycling conditions (BigDye_Kit _Fast): 96 °C for 1 min, 25 cycles of 96 °C for 10 s, 50 °C for 5 s and 60 °C for 75 s, and hold at 4 °C. PCR products were purified with a BigDye XTerminator Purification Kit (Thermo Fisher Scientific) and subsequently sequenced on a 3500 Genetic Analyzer (Thermo Fisher Scientific). The data were analyzed by Sequencing Analysis Software v5.4 (Thermo Fisher Scientific) [18, 25, 33].

### Validation by NGS

Sequencing libraries were prepared using the Ion AmpliSeq Library Kit Plus (Thermo Fisher Scientific) as previously described [17, 19, 23, 28, 34]. Briefly, multiplex PCR was performed using the Ion AmpliSeq™ Cancer Hotspot Panel v2 (Thermo Fisher Scientific), which targets the hotspot regions of 50 oncogenes and tumour suppressor genes [17–28, 32–37]. PCR products were partially digested with FuPa reagent and subsequently ligated to adaptors and barcodes using the Ion Xpress Barcode Adapters Kit (Thermo Fisher Scientific). The ligated library was purified with Agencourt AMPure XP reagent (Beckman Coulter, Brea, CA), and the library concentration was determined using an Ion Library Quantitation Kit (Thermo Fisher Scientific). Each library was diluted and the same amount of each was pooled. Emulsion PCR and chip loading was performed on an Ion Chef with the Ion PGM Hi-Q View Chef Kit. Sequencing was performed using an Ion PGM Hi-Q View Sequencing Kit on the Ion PGM (Thermo Fisher Scientific). Variant calling and annotation were performed using an Ion Reporter Server System (Thermo Fisher Scientific). We identified nonsynonymous mutations with the AmpliSeq CHPv2 single sample workflow (version 5.10) and used the following filtering parameters: (i) a minimum count of ≥10 for mutant allele reads, (ii) coverage depth ≥ 20 at the somatic variant site, (iii) variant allele faction ≥5%, and *p*-value cut-off of 0.05, and (iv) variants present in the dbSNP database (version 138) were filtered out (UCSC Common SNPs = Not In) [24, 27]. Binary SAM (BAM) files were visualized by Ion Reporter™ Genomic Viewer.

### Sensitivity determination

To examine sensitivity testing experiments, TaqMan™ Control Genomic DNA (human) (Thermo Fisher Scientific) was spiked with different amounts of Horizon Tru-Q 7 (1.3% Tier) Reference Standard (Horizon Discovery, Cambridge, UK) harboring engineered mutations. The mixtures represented 1–33% and 0.4–12% variant allele fraction range in KRAS at codon 12/13 and BRAF at codon 600, respectively. The total number of DNA molecules was kept in constant.

## Results

### *KRAS* and *NRAS* mutations detected by PCR-rSSO

**Fig. 1:**
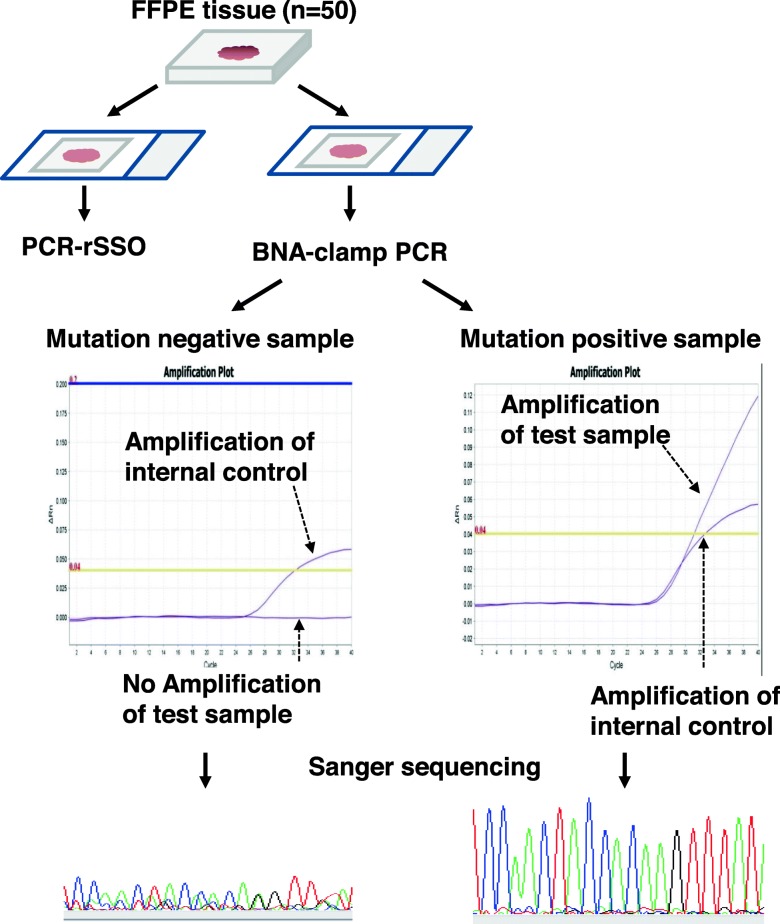
Experimental design. We tested FFPE samples from 50 patients with colorectal cancer. Tissue sections were subjected to PCR-rSSO and BNA-clamp PCR. BNA-clamp PCR involved real-time PCR and amplified the mutated allele. The amplification plot was verified using mutation-positive samples and an internal control was amplified to confirm assay integrity. PCR products from BNA-clamp PCR were subsequently analyzed by Sanger sequencing to confirm the nucleotide changes

We analyzed FFPE tissues from 50 patients with colorectal cancer. DNA was extracted from sections and subjected to PCR-rSSO (Fig. 1). Of 50 samples, at least one mutation in either *KRAS* or *NRAS* was detected in 31 samples (Table 1). Twenty five mutations were found in *KRAS* (17 at codon 12, 5 at codon 13, 1 at codon 61, and 2 at codon 146). Six mutations were detected in *NRAS* (2 at codon 12, 1 at codon 13, and 3 at codon 61).

**Table 1:** Comparison of *KRAS* and *NRAS* mutation status determined by PCR-rSSO, BNA-clamp PCR and NGS

	PCR-rSSO		BNA-clamp PCR		Deep sequencing by NGS	
Sample No.	Nucleotide changes	Deduced amino acid changes	Nucleotide changes	Deduced amino acid changes	Nucleotide changes	Deduced amino acid changes
#1	Wild-type	Wild-type	c.180_181delTCinsAA	KRAS Q61K	c.180_181delTCinsAA	KRAS Q61K
#2	c.34G > T	NRAS G12C	Wild-type	Wild-type	Wild-type	Wild-type
#3	c.436G > A	KRAS A146T	c.436G > A	KRAS A146T	c.436G > A	KRAS A146T
	Wild-type	Wild-type	c.35G > A	NRAS G12D	c.35G > A	NRAS G12D
#4	c.35G > C	KRAS G12A	c.35G > C	KRAS G12A	c.35G > C	KRAS G12A
#5	c.35G > C	KRAS G12A	c.35G > C	KRAS G12A	c.35G > C	KRAS G12A
#6	c.35G > C	KRAS G12A	c.35G > C	KRAS G12A	c.35G > C	KRAS G12A
#7	c.34G > T	KRAS G12C	c.34G > T	KRAS G12C	c.34G > T	KRAS G12C
#8	c.35G > A	KRAS G12D	c.35G > A	KRAS G12D	c.35G > A	KRAS G12D
#9	c.35G > A	KRAS G12D	c.35G > A	KRAS G12D	c.35G > A	KRAS G12D
#10	c.35G > A	KRAS G12D	c.35G > A	KRAS G12D	c.35G > A	KRAS G12D
#11	c.35G > A	KRAS G12D	c.35G > A	KRAS G12D	c.35G > A	KRAS G12D
#12	c.35G > A	KRAS G12D	c.35G > A	KRAS G12D	c.35G > A	KRAS G12D
#13	c.35G > A	KRAS G12D	c.35G > A	KRAS G12D	c.35G > A	KRAS G12D
#14	c.35G > A	KRAS G12D	c.35G > A	KRAS G12D	c.35G > A	KRAS G12D
#15	c.35G > A	KRAS G12D	c.35G > A	KRAS G12D	c.35G > A	KRAS G12D
#16	c.34G > A	KRAS G12S	c.34G > A	KRAS G12S	c.34G > A	KRAS G12S
#17	c.34G > A	KRAS G12S	c.34G > A	KRAS G12S	c.34G > A	KRAS G12S
#18	c.35G > T	KRAS G12 V	c.35G > T	KRAS G12 V	c.35G > T	KRAS G12 V
#19	c.35G > T	KRAS G12 V	c.35G > T	KRAS G12 V	c.35G > T	KRAS G12 V
#20	c.35G > T	KRAS G12 V	c.35G > T	KRAS G12 V	c.35G > T	KRAS G12 V
#21	c.38G > A	KRAS G13D	c.38G > A	KRAS G13D	c.38G > A	KRAS G13D
#22	c.38G > A	KRAS G13D	c.38G > A	KRAS G13D	c.38G > A	KRAS G13D
#23	c.38G > A	KRAS G13D	c.38G > A	KRAS G13D	c.38G > A	KRAS G13D
#24	c.38G > A	KRAS G13D	c.38G > A	KRAS G13D	c.38G > A	KRAS G13D
#25	c.38G > A	KRAS G13D	c.38G > A	KRAS G13D	c.38G > A	KRAS G13D
#26	c.182A > T	KRAS Q61L	c.182A > T	KRAS Q61L	c.182A > T	KRAS Q61L
#27	c.436G > A	KRAS A146T	c.436G > A	KRAS A146T	c.436G > A	KRAS A146T
#28	c.35G > A	NRAS G12D	c.35G > A	NRAS G12D	c.35G > A	NRAS G12D
#29	c.38G > T	NRAS G13 V	c.38G > T	NRAS G13 V	c.38G > T	NRAS G13 V
#30	c.181C > A	NRAS Q61K	c.181C > A	NRAS Q61K	c.181C > A	NRAS Q61K
#31	c.181C > A	NRAS Q61K	c.181C > A	NRAS Q61K	c.181C > A	NRAS Q61K
#32	c.182A > G	NRAS Q61R	c.182A > G	NRAS Q61R	c.182A > G	NRAS Q61R
#33	NA	NA	c.1799 T > A	BRAF V600E	c.1799 T > A	BRAF V600E
#34	NA	NA	c.1799 T > A	BRAF V600E	c.1799 T > A	BRAF V600E
#35	NA	NA	c.1799 T > A	BRAF V600E	c.1799 T > A	BRAF V600E
#36–50	Wild-type	Wild-type	Wild-type	Wild-type	Wild-type	Wild-type

*NA* not applicable

### *KRAS* and *NRAS* mutations detected by BNA-clamp PCR with Sanger sequencing

We also analyzed the 50 samples for *KRAS* and *NRAS* mutations using BNA-clamp PCR (Fig. 1). Of 50 samples, amplification plots of 26 samples reached to threshold, but those of five samples did not reached (Supplemental Table 1). In these five samples, we detected either *KRAS* and/or *NRAS* mutations by Sanger sequencing using PCR product of BNA-clamp PCR. Therefore, at least one mutation in *KRAS* or *NRAS* was identified in 31 samples (Table 1). Twenty six mutations were found in *KRAS* (17 at codon 12, 5 at codon 13, 2 at codon 61, and 2 at codon 146). Six mutations were identified in *NRAS* (2 at codon 12, 1 at codon 13, and 3 at codon 61). In one sample (sample #3), two mutations (*KRAS* at codon 146 and *NRAS* at codon 12*)* were identified (Table 1).

### Comparison of identified mutations by PCR-rSSO and BNA-clamp PCR with Sanger sequencing

We next compared the *KRAS* and *NRAS* mutation status identified by the two different methods. There was 94% (47/50) concordance between PCR-rSSO and BNA-clamp PCR with Sanger sequencing. Positive percent agreement was 94% (29/31) and negative percent agreement was 95% (18/19). In three samples (sample #1-#3 in Table 1), there were discordant results. In sample #1, *KRAS* c.180_181delTCinsAA (p.Q61K) was detected by BNA-clamp PCR, but not by PCR-rSSO (Table 1). In sample #2, *NRAS* c.34G > T (p.G12C) was detected by PCR-rSSO, but not by BNA-clamp PCR. In sample #3, BNA-clamp PCR with Sanger sequencing identified two nucleotide changes, *KRAS* c.436G > A (p.A146T) and *NRAS* c.35G > A (p.G12D), whereas PCR-rSSO identified only one nucleotide change, *KRAS* c.436G > A.

### Validation by NGS

To validate the results from the two methods, we subjected the three discordant samples (samples #1-#3) as well as 47 concordant samples (#4-#50) to NGS covering hotspot mutations of 50 cancer-associated genes. NGS yielded the sufficient sequencing reads mapped on the target regions (mean: 97%) and an average base coverage depth on targeted reference region (mean: 10,849-fold) (Table 2).

**Table 2:** Quality and coverage depth of next generation sequencing

Sample	Mapped reads	On target	Mean depth	Uniformity
#1	3,896,308	98%	17,984	98%
#2	2,686,903	98%	12,262	96%
#3	1,202,870	98%	5057	80%
#4	2,101,697	98%	9467	92%
#5	2,069,593	94%	8935	99%
#6	2,623,488	98%	11,775	90%
#7	2,950,289	97%	13,056	99%
#8	1,834,917	98%	8227	87%
#9	3,270,772	98%	14,836	96%
#10	3,957,906	99%	18,101	100%
#11	2,980,026	98%	13,450	100%
#12	1,518,770	98%	6864	86%
#13	2,569,739	91%	10,930	100%
#14	3,089,053	99%	14,112	100%
#15	3,471,557	99%	15,934	100%
#16	2,385,272	98%	10,832	90%
#17	2,990,670	99%	14,066	100%
#18	2,803,954	98%	12,871	100%
#19	1,818,393	97%	8086	99%
#20	649,510	97%	2794	92%
#21	3,236,156	99%	14,950	99%
#22	2,215,854	98%	9819	100%
#23	2,600,289	99%	11,942	98%
#24	2,487,267	97%	11,077	96%
#25	2,127,006	98%	9555	85%
#26	1,227,270	68%	3341	75%
#27	2,386,360	95%	10,366	99%
#28	3,042,162	98%	13,665	95%
#29	3,321,952	98%	14,756	96%
#30	2,905,123	98%	12,934	92%
#31	2,437,114	98%	10,958	90%
#32	3,031,339	97%	13,566	98%
#33	2,819,335	98%	12,766	93%
#34	2,335,610	98%	10,532	82%
#35	3,136,401	97%	14,138	98%
#36	2,583,625	97%	11,885	99%
#37	1,987,561	96%	8886	97%
#38	1,161,909	95%	5181	98%
#39	1,592,153	98%	7337	97%
#40	1,421,966	98%	6490	91%
#41	1,716,683	98%	7854	93%
#42	2,121,085	99%	9920	97%
#43	1,970,851	98%	8928	99%
#44	2,510,670	99%	11,608	99%
#45	1,398,962	98%	6231	99%
#46	2,872,517	97%	13,063	98%
#47	1,760,828	97%	7928	97%
#48	2,259,832	97%	9915	90%
#49	1,948,999	98%	8921	99%
#50	3,134,126	98%	14,287	96%

Mapped reads: number of sequencing reads that were mapped to the human genome

On target: percentage of mapped reads that were aligned over the target region

Mean depth: Average base coverage depth over all bases targeted in the reference

Uniformity: percentage of target bases covered by at least 0.2x the average base read depth

NGS detected *KRAS* c.180_181delTCinsAA (p.Q61K) in sample #1, *KRAS* c.205G > A (p.D69N) in sample #2 and *KRAS* c.436G > A (p.A146T) and *NRAS* c.35G > A (p.G12D) mutations in sample #3 (Table 3). Although *NRAS* c.34G > T (p.G12C) was identified by PCR-rSSO in sample #2, this mutation was not identified by NGS. Both BNA-clamp PCR and PCR-rSSO methods did not detect *NRAS* p.D69N in sample #2, because this variant was not covered by either method (Table 3). Furthermore, remaining 47 samples were concordant among PCR-rSSO, BNA-clamp PCR with Sanger sequencing and NGS. Overall, the NGS results were concordant with those of BNA-clamp PCR with Sanger sequencing. These results indicate that *KRAS* and *NRAS* mutations were accurately detected by BNA-clamp PCR with Sanger sequencing.

**Table 3:** Mutations in discordant samples identified by next generation sequencing using a panel of 50 cancer-associated genes

Sample No.	Position	Reference	Variant	Gene	Nucleotide changes	Deduced amino acid changes	VAF (%)	coverage
#1	chr12:25380277	GA	TT	***KRAS***	**c.180_181delTCinsAA**	**Q61K**	23.5	1982
#2	chr1:115256506	C	T	*NRAS*	c.205G > A	D69N	17.5	1995
	chr14:105246470	C	T	*AKT1*	c.130G > A	D44N	5.5	2000
	chr17:7578431	G	A	*TP53*	c.499C > T	Q167*	21.1	1970
	chr19:1223030	C	T	*STK11*	c.967C > T	P323S	5.1	2000
	chr19:1223054	C	T	*STK11*	c.991C > T	R331W	5.1	1997
#3	chr12:25378562	C	T	***KRAS***	**c.436G > A**	**A146T**	28.3	1161
	chr1:115258747	C	T	***NRAS***	**c.35G > A**	**G12D**	9.1	1998
	chr3:37067240	T	A	*MLH1*	c.1151 T > A	V384D	21.6	1998
	chr5:112173917	C	T	*APC*	c.2626C > T	R876*	16.1	2000
	chr5:112175589	C	T	*APC*	c.4298C > T	P1433L	7.4	2000
	chr13:49033902	T	C	*RB1*	c.2039 T > C	I680T	6.5	1628
	chr17:7577551	C	A	*TP53*	c.730G > T	G244C	12.6	2000
	chr17:7578479	G	A	*TP53*	c.451C > T	P151S	18.0	2000
	chr19:1223125	C	G	*STK11*	c.1062C > G	F354 L	41.2	818

Bold text indicates mutations covered by BNA-clamp and PCR-rSSO

*VAF* variant allele frequency

**Fig. 2:**
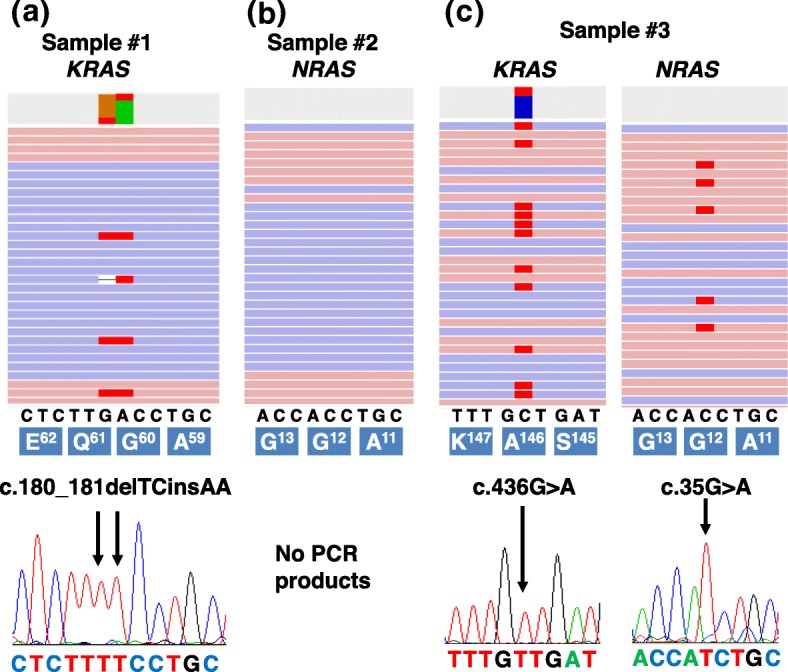
Discordant results were validated by NGS and Sanger sequencing. **a**-**c** Representative images of read alignments (BAM files) of sample #1 (**a**), #2 (**b**) and #3 (**c**) were visualized with Ion Reporter Genome Viewer (upper images). PCR products produced by BNA-clamp PCR were purified and used as templates for Sanger sequencing. Sequencing chromatograms show the mutations in each sample (lower image). Arrows indicate the position of the mutations. **a** In sample #1, *KRAS* p.Q61K was detected by NGS. At this site, multi-nucleotide variants (c.180_181delTCinsAA) existed in codons 60 and 61. **b** In sample #2, there was no apparent variant at codon 12 of *NRAS*. We did not observe an amplification plot signal by real-time PCR and obtained no visible PCR product for subsequent Sanger sequencing analysis. **c** Two different mutations (*NRAS* p.G12D and *KRAS* p.A146T) were observed in sample #3

To examine the reasons for the discordant results, we checked the NGS results and BNA-clamp PCR with Sanger sequencing. In sample #1, harboring *KRAS* p.Q61K, there were multi-nucleotide variants (c.180_181delTCinsAA) across codon 60 and 61 (Fig. 2 and Table 3). The nucleotide change at codon 60 (GGT > GGA) led to a synonymous mutation (*KRAS* p.G60G). Because PCR-rSSO detects only perfectly-matched single nucleotide variants, it did not detect the multi-nucleotide variants [29]. In sample #2, *NRAS* c.34G > T (p.G12C) was not identified by either BNA-clamp PCR or NGS (Fig. 2 and Additional file 2: Figure S2).

### *BRAF* mutations

*BRAF* mutations occur in approximately 10% of colorectal cancers and are associated with resistance to anti-EGFR therapy [9]. PCR-rSSO using the MEBGEN™ RASKET kit did not cover *BRAF* mutations. However, *BRAF* mutations were detected by BNA-clamp PCR and NGS in 3 of the 50 (6%) samples (sample #33–35), which were wild-type for *KRAS* and *NRAS* (Table 1).

### Turnaround time

We assessed the turnaround time of PCR-rSSO and BNA-clamp PCR methods. It takes approximately 4.5 h with PCR-rSSO, whereas 2 h with BNA-clamp PCR. Because PCR-rSSO method uses DNA-probe for hybridization to detect mutated DNA, it takes more long time for hybridize reaction. Contrary, BNA-clamp method contains mixed BNA-clamping probe and primers in reaction reagent and needs one-step real-time PCR reaction for detecting mutation. If PCR product of BNA-clamp PCR is analyzed by Sanger sequencing, it additionally takes about 3 h. Combined with BNA-clamp PCR and Sanger sequencing, it takes a total of 5 h.

## Discussion

In this study, we compared mutation detection in *KRAS* and *NRAS* genes between PCR-rSSO and BNA-clamp PCR with Sanger sequencing. Overall, the concordance rate was 94% (47/50 samples) between the two methods. However, there were three discordant results which were further analyzed by NGS with high-depth coverage. The NGS results were consistent with the results of BNA-clamp PCR with Sanger sequencing. Our results demonstrated the BNA-clamp PCR method with Sanger sequencing have high accuracy for the detection of *KRAS*, *NRAS* and *BRAF* mutations in colorectal cancer.

According to manufacturer’s instructions, the limit of detections of both BNA-clamp PCR and PCR-rSSO methods were 1–5%. We confirmed the sensitivity by diluted experiment of BNA-clamp PCR (Additional file 3: Figure S3). Although the performance of sensitivity is comparable, BNA-clamp PCR with Sanger sequencing has several advantages. First, it requires only standard clinical laboratory equipment (e.g. a real-time PCR and capillary sequencer). We could qualitatively evaluate the presence of mutations by real-time PCR. The BNA probe binds to wild-type template DNA and inhibits its PCR amplification, whereas mutated alleles are selectively amplified during real-time PCR (Additional file 1: Figure S1). Second, BNA-clamp PCR can analyze multi-nucleotide variants within *KRAS*, *NRAS* and *BRAF* genes. Third, BNA-clamp PCR is a simple method with a short turnaround time. PCR-rSSO takes 4.5 h per run: PCR reaction (2.3 h), hybridization (1.4 h) and detection of fluorescence with dedicated equipment (0.8 h). BNA-clamp PCR takes only 2 h per run: preparation of PCR master mix (0.5 h) and real time PCR reaction (1.5 h). Even when PCR products of BNA-clamp PCR were investigate by the Sanger sequencing, it takes about 30 min longer compared to PCR-rSSO. Fourth, the running cost is lower than PCR-rSSO; it costs approximately 50 USD per BNA-clamp PCR reaction compared with approximately 178 USD per PCR-rSSO reaction. It costs additionally 20 USD when Sanger sequencing is conducted.

We examined the three discordant results between PCR-rSSO with Sanger sequencing and BNA-clamp methods. The reason for discordance was explained in only sample #1. The PCR-rSSO method detects perfectly-matched mutated alleles in tumor samples; therefore, it missed the *KRAS* multi-nucleotide variant, c.180_181delTCinsAA, and reported this site as wild type (Table 1). A previous report also showed PCR-rSSO could not detect *KRAS* p.G12C because of a multi-nucleotide variant at codon 11 (c.33_34delTGinsCT) [29]. In sample #2, PCR-rSSO reported an *NRAS* c.34G > T (p.G12C) mutation but both BNA-clamp PCR and deep sequencing did not. To exclude the possibility that the variant calling filtered out the *NRAS* mutation, we further visualized BAM data using Ion Reporter™ Genomic Viewer. However, we could not confirm the corresponding mutated reads in *NRAS* (Additional file 2: Figure S2). In sample #3, both BNA-clamp PCR and NGS detected the *NRAS* (c.35G > A) p.G12D mutation at 9.1% variant allele fraction. Although the reagent kit included a perfectly-matched probe corresponding to *NRAS* c.35G > A (p.G12D), PCR-rSSO did not detect this mutation.

The reasons for these two discordant results remain unclear, but one possible explanation is tumor heterogeneity. We prepared sections from patient FFPE tissues and each method analyzed different sections (Fig. 1). PCR-rSSO was conducted by a commercial laboratory on tissue without microdissection. In contrast, BNA-clamp PCR with Sanger sequencing and NGS used microdissected tumor tissue samples (Arcturus; Thermo Fisher Scientific, MA, USA) [19, 38]. Alternatively, there may be differences in quality control between laboratories. Our laboratory (GAC-Genome Analysis Center) sent specimens to the College of American Pathologists (CAP) for proficiency testing and achieved a 100% match.

BNA-clamp PCR has limitations. Although we could identify the presence of mutations in samples by real-time PCR, Sanger sequencing is needed to determine the nucleotide changes. In addition, the kit contains nine primer/probe mixtures, which are designed to interrogate *KRAS*, *NRAS* and *BRAF* exons. When a small number of samples (less than eight) is to be tested, they can be analyzed in one reaction in a 96-well format. When more samples need to be analyzed, several real-time PCRs are needed. Therefore, small to medium numbers of samples are suitable for analysis by BNA-clamp PCR. In one sample (#3), the amplification plot did not reached to threshold line by BNA-clam PCR, though variant allele fraction of *KRAS* A146T and *NRAS* G12D by the NGS were 28.3 and 9.1%, respectively. Although the precise reason is not unclear, there are several possibilities. One is the quality of the DNA from FFPE tissue. Fragmented FFPE DNA may be not effectively amplified by BNA-clamp PCR in this sample. Second possibility may be the difference of DNA polymerase enzyme used for reaction. In general, high-activity and fidelity DNA polymerase is used in NGS library construction. The PCR amplification efficiency may be different between BNA-clamp PCR and NGS. According to these possibilities, NGS could detect the mutation nevertheless less quality of DNA was used as long as targeted regions was successfully amplified and NGS library was constructed.

Overall, we confirmed the clinical utility of BNA-clamp PCR for detecting *KRAS*, *NRAS* and *BRAF* mutations in colorectal cancers. This less time-consuming and less laborious method can enable precision medicine to be offered to patients with metastatic colorectal cancers, such as anti-EGFR therapy. Furthermore, circulating tumor DNA (ctDNA) was shed into the blood stream and body fluids, called as liquid biopsy. The detection of ctDNA is useful for monitoring tumor recurrence, predicting treatment effect and detecting drug-resistant mutation in patients with colorectal cancer. BNA-clamp PCR would be one of the candidate methods for detecting rare mutations in liquid biopsy in a clinical laboratory.

## Conclusions

In this study, we estimated the performance of BNA-clamp PCR with Sanger sequencing method to detect *KRAS*, *NRAS* and *BRAF* mutations in colorectal cancers and compared the results from PCR-rSSO and NGS. BNA-clamp PCR accurately detected *KRAS*, *NRAS* and *BRAF* mutations in patients with colorectal cancer. Genetic testing by BNA-clamp PCR with Sanger sequencing has potential to be used in routine clinical practice for the selection of appropriate patients for anti-EGFR therapy.

## Supplementary information

**Additional file 1: Figure S1.** Principle of BNA-clamp PCR. Forward and reverse primers amplify the targeted mutation. A BNA probe binds to the wild-type allele but not to the mutated allele. The BNA probe selectively inhibits PCR amplification of the wild-type allele. F, fluorescence; Q, quencher.

**Additional file 2: Figure S2.** Sequence reads were visualized by Ion Reporter Genome Viewer. Representative images of read alignments (BAM files) of sample #2 were visualized with Ion Reporter Genome Viewer. There are no mutated reads corresponding to *NRAS* p.G12C (c.34G > T: chr1:115,258,748) in the next generation sequencing data.

**Additional file 3: Figure S3.** Amplification plot of dilution experiment by BNA-clamp PCR method. Wild-type control DNA was spiked in the Tru-Q 7 (1.3% Tier) Reference Standard. BNA-clamp PCR was performed using serial dilution DNA. (A) DNA containing *KRAS* mutation at codon 12/13 (dilution range: 1–33% variant allele fraction) and (B) *BRAF* mutation at codon 600 (dilution range: 0.4–12% variant allele fraction).

**Additional file 4: Table S1.** Threshold cycle values of real-time PCR using BNA-clamp PCR method.

## Data Availability

The datasets used and/or analysed during the current study are available from the corresponding author on reasonable request.

## Abbreviations

BAM: Binary SAM
BNA: Bridged nucleic acid
BNA-clamp PCR: bridged nucleic acid-clamp real-time PCR
BRAF: v-raf murine sarcoma viral oncogene homolog B1
CAP: College of American Pathologists
EGFR: Epidermal growth factor receptor
FFPE: Formalin-fixed paraffin embedded
KRAS: Kirsten rat sarcoma viral oncogene homolog
NGS: Next generation sequencing
NRAS: Neuroblastoma RAS viral oncogene homolog
PCR-rSSO: PCR–reverse sequence-specific oligonucleotide probe
SA-PE: Phycoerythrin-labeled streptavidin
UNG: Uracil-N-glycosylase
