# Supplementary figures and images for: Accurate detection of KRAS, NRAS and BRAF mutations in metastatic colorectal cancers by bridged nucleic acid-clamp real-time PCR
Source: BMC Med Genomics. 2019 Nov 11;12:162. doi: 10.1186/s12920-019-0610-8 (PMC6849194; doi:10.1186/s12920-019-0610-8)

## Supplemental Figure 1

### Wild-type allele

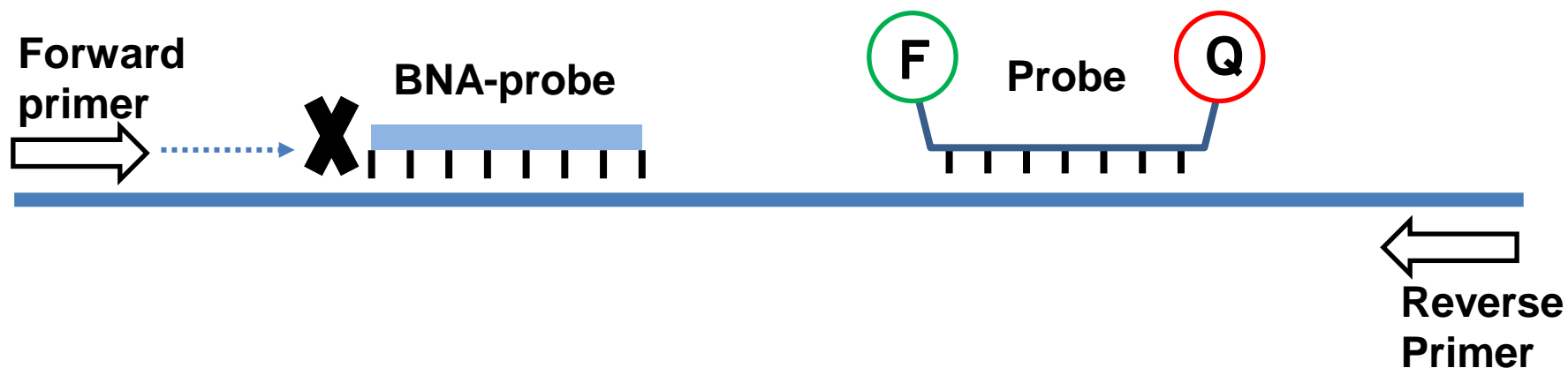

### Mutated allele

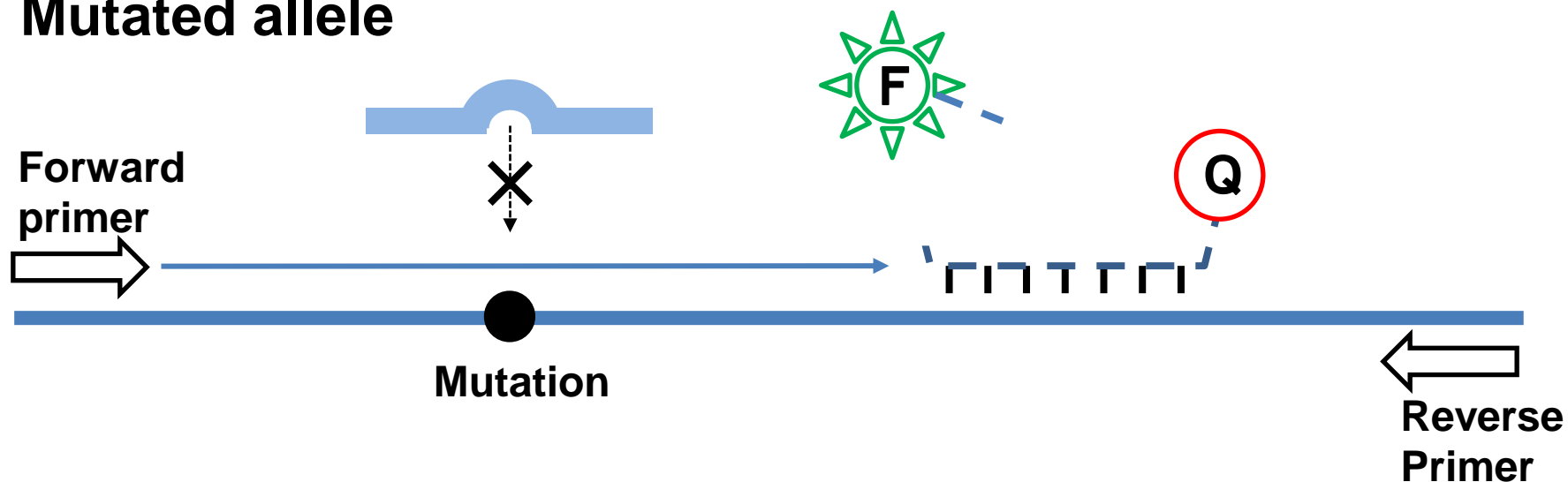

Supplement: Supplementary file 1 — Additional file 1: Figure S1. Principle of BNA-clamp PCR. Forward and reverse primers amplify the targeted mutation. A BNA probe binds to the wild-type allele but not to the mutated allele. The BNA probe selectively inhibits PCR amplification of the wild-type allele. F, fluorescence; Q, quencher. [file 12920_2019_610_MOESM1_ESM.pdf]

# Supplemental Figure 2

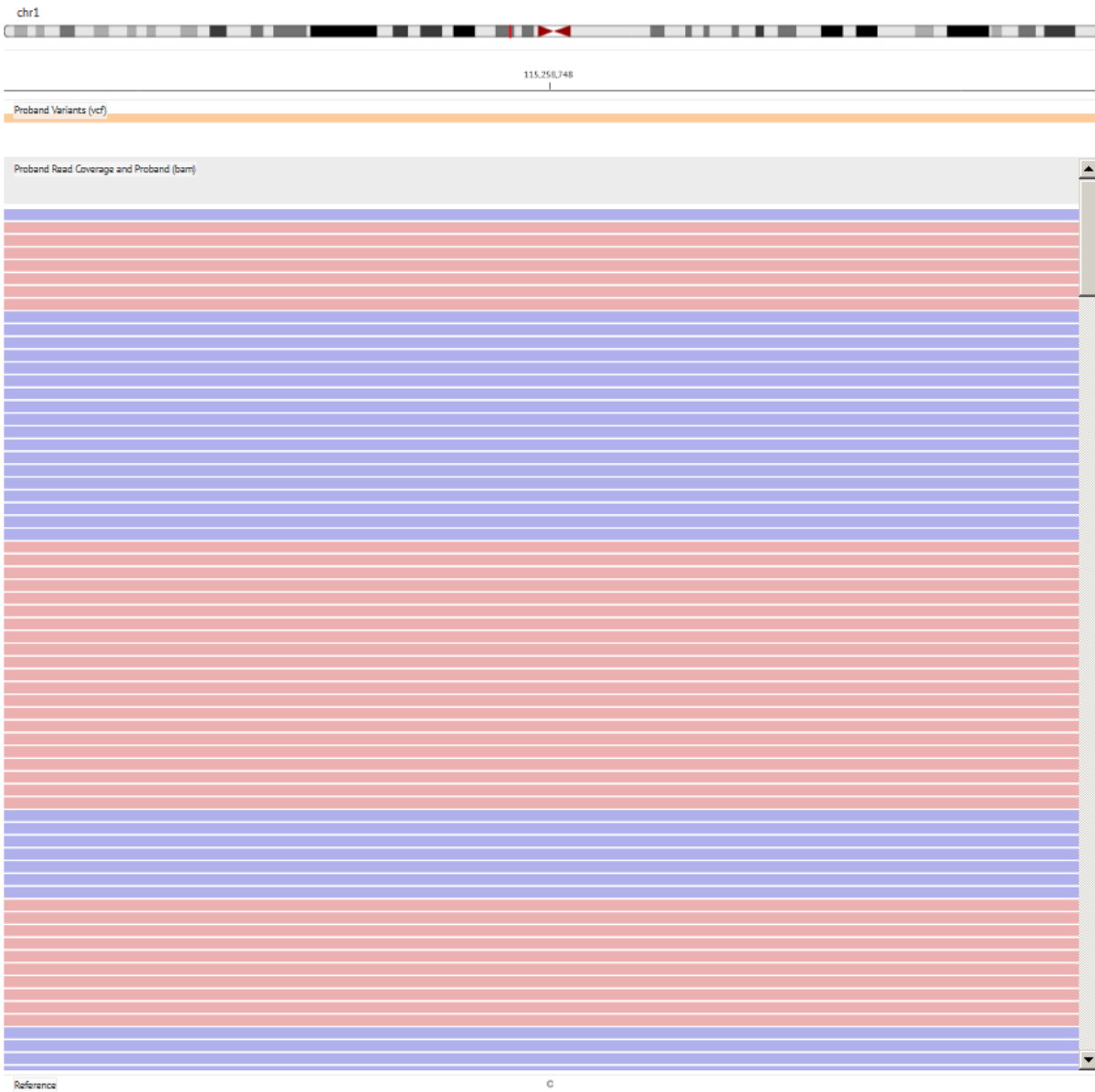

Supplement: Supplementary file 2 — Additional file 2: Figure S2. Sequence reads were visualized by Ion Reporter Genome Viewer. Representative images of read alignments (BAM files) of sample #2 were visualized with Ion Reporter Genome Viewer. There are no mutated reads corresponding to NRAS p.G12C (c.34G > T: chr1:115,258,748) in the next generation sequencing data. [file 12920_2019_610_MOESM2_ESM.pdf]

(A)

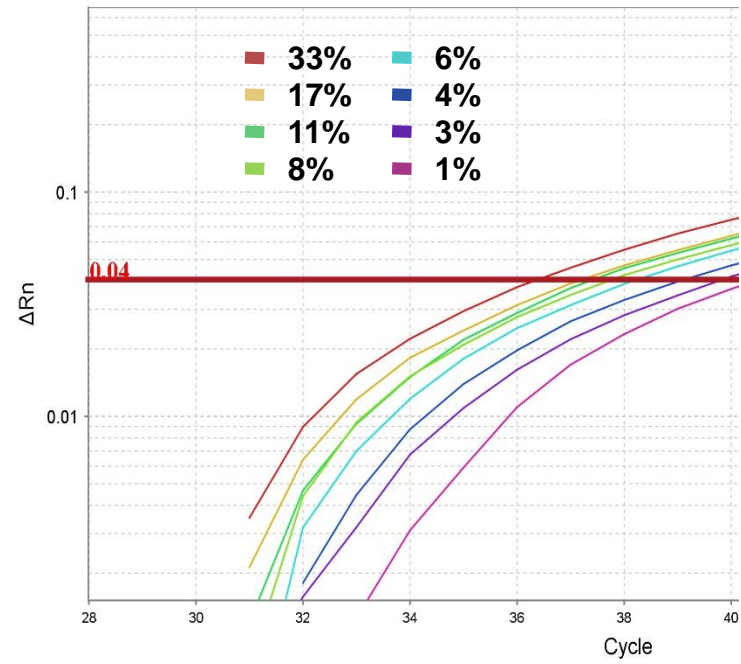

(B)

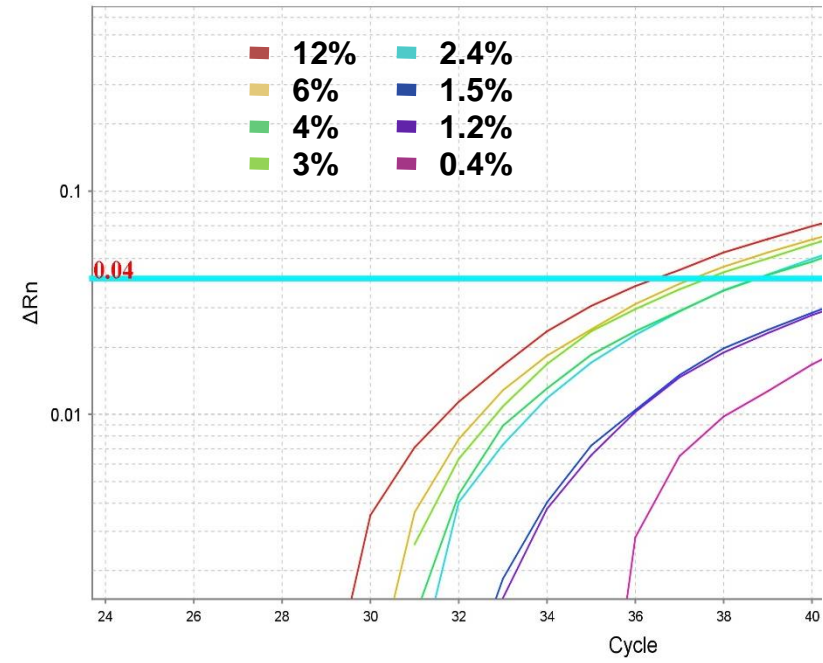

Supplement: Supplementary file 3 — Additional file 3: Figure S3. Amplification plot of dilution experiment by BNA-clamp PCR method. Wild-type control DNA was spiked in the Tru-Q 7 (1.3% Tier) Reference Standard. BNA-clamp PCR was performed using serial dilution DNA. (A) DNA containing KRAS mutation at codon 12/13 (dilution range: 1–33% variant allele fraction) and (B) BRAF mutation at codon 600 (dilution range: 0.4–12% variant allele fraction). [file 12920_2019_610_MOESM3_ESM.pdf]
